# Comprehensive transcriptomic analysis unveils macrophage-associated genes for establishing an abdominal aortic aneurysm diagnostic model and molecular therapeutic framework

**DOI:** 10.1186/s40001-024-01900-w

**Published:** 2024-06-12

**Authors:** Zhen Wu, Weiming Yu, Jie Luo, Guanghui Shen, Zhongqi Cui, Wenxuan Ni, Haiyang Wang

**Affiliations:** 1 Department of Vascular and Interventional Surgery, The First Affiliated Hospital of Harbin Medical University, Harbin, 150001 Heilongjiang China; 2 Department of Clinical Laboratory, Shanghai Tenth People’s Hospital, School of Medicine, Tongji University, Shanghai, 200072 China; 3 General Surgery, Thyroid Surgery, Sun Yat-sen Memorial Hospital, Sun Yat-sen University, Guangzhou, 510000 Guangdong China

**Keywords:** Abdominal aortic aneurysm, Atherosclerosis, Single-cell sequencing, Macrophage marker genes, Machine learning

## Abstract

**Background:**

Abdominal aortic aneurysm (AAA) is a highly lethal cardiovascular disease. The aim of this research is to identify new biomarkers and therapeutic targets for the treatment of such deadly diseases.

**Methods:**

Single-sample gene set enrichment analysis (ssGSEA) and CIBERSORT algorithms were used to identify distinct immune cell infiltration types between AAA and normal abdominal aortas. Single-cell RNA sequencing data were used to analyse the hallmark genes of AAA-associated macrophage cell subsets. Six macrophage-related hub genes were identified through weighted gene co-expression network analysis (WGCNA) and validated for expression in clinical samples and AAA mouse models. We screened potential therapeutic drugs for AAA through online Connectivity Map databases (CMap). A network-based approach was used to explore the relationships between the candidate genes and transcription factors (TFs), lncRNAs, and miRNAs. Additionally, we also identified hub genes that can effectively identify AAA and atherosclerosis (AS) through a variety of machine learning algorithms.

**Results:**

We obtained six macrophage hub genes (IL-1B, CXCL1, SOCS3, SLC2A3, G0S2, and CCL3) that can effectively diagnose abdominal aortic aneurysm. The ROC curves and decision curve analysis (DCA) were combined to further confirm the good diagnostic efficacy of the hub genes. Further analysis revealed that the expression of the six hub genes mentioned above was significantly increased in AAA patients and mice. We also constructed TF regulatory networks and competing endogenous RNA networks (ceRNA) to reveal potential mechanisms of disease occurrence. We also obtained two key genes (ZNF652 and UBR5) through a variety of machine learning algorithms, which can effectively distinguish abdominal aortic aneurysm and atherosclerosis.

**Conclusion:**

Our findings depict the molecular pharmaceutical network in AAA, providing new ideas for effective diagnosis and treatment of diseases.

**Supplementary Information:**

The online version contains supplementary material available at 10.1186/s40001-024-01900-w.

## Introduction

An abdominal aortic aneurysm (AAA) is a permanent dilatation of the aorta with a diameter of over 3 cm, which is susceptible to rupture and causes life-threatening haemorrhage. According to recent research, the global prevalence of AAA among persons aged 30–79 years is 0.92%, with an overall mortality exceeding 80% [1, 2]. AAA pathogenesis is complex and involves elastin degradation, collagen remodelling, smooth muscle cell apoptosis, and inflammatory cells [3]. Among these, the inflammatory process plays a crucial role in AAA development, leading to subsequent aortic wall remodelling. Currently, there are no effective drug-based therapies that can inhibit or prevent AAA rupture [4, 5]. Surgical repair, including endovascular aortic repair (EVAR) and open surgical repair, is an effective option for AAA management [6]. However, the threshold of aortic diameter used for elective surgical repairs is > 5.5 cm for men and > 5.0 cm for women [6, 7], whereas patients with smaller AAA are left untreated and recommended for surveillance. Nevertheless, ~ 10% of AAA rupture at a size smaller than the threshold, and it is challenging to perform continuous surveillance of AAA because of the nonlinearity and unpredictability of the expansion rate [8–10]. Analysing heterogeneous molecules within the aneurysm and normal abdominal aorta will facilitate the investigation and understanding of AAA pathogenesis to establish a diagnostic model and identify effective therapeutic targets for timely intervention.

The infiltration of immune cells into the abdominal aortic wall is an important focus of investigation because chronic inflammation caused by these cells is a key feature and driver of AAA [11]. Macrophages that accumulate in the aortic wall play an important role in the occurrence and development of AAA. They are involved in extracellular matrix remodelling, the promotion and resolution of inflammation, and tissue healing. This sustained inflammatory response leads to dilation of the aorta and subsequent formation of an aortic aneurysm. Reportedly, inhibition of the inflammatory response can effectively limit the development and progression of AAA [12, 13]. Notably, patients with AAA frequently have atherosclerotic changes in their coronary wall [14]. There may be a connection between AAA and atherosclerosis, given that patients with AAA often have atherosclerosis, such as coronary artery disease and peripheral artery disease (PAD) [15–17]. Etiologically, these two diseases share similar risk factors, such as increasing age, male sex, smoking, hypertension, obesity, and low HDL cholesterol levels [2, 18]. In terms of pathological manifestations, the lesion sites in both diseases show similar immune and inflammatory cell infiltration features, albeit in different vascular layers [19]. One theory suggests that atherosclerosis is a precursor of AAA because changes in the aortic wall caused by atherosclerosis are the pathological basis for AAA formation [14]. Considering this, we aimed to identify markers that could distinguish between the two diseases and screen individuals who were on the verge of progressing to AAA from those with atherosclerosis.

In this study, we used the GEO online public database for AAA single-cell sequencing analysis, WGCNA analysis, and differential expression analysis to construct a six-genes AAA diagnostic model, which has high clinical value in diagnosing AAA. To enhance the persuasiveness of bioinformatics predictions, we validated the expression of the aforementioned genes in AAA mice tissue and clinical patients’ serum and evaluated the diagnostic efficacy of AAA. Considering the important value of the six genes above mentioned, we further constructed TF and lncRNA–miRNA–mRNA molecular networks to reveal potential molecular regulatory mechanisms. Finally, we constructed a diagnostic model containing two genes through various machine learning methods, which can effectively distinguish AAA and AS, which is of great value for early warning of AAA. In addition, we also discussed the relationship between risk model and immune cell infiltration to better understand the potential molecular immune processes in disease progression. In summary, our research provides new insights for the effective diagnosis and clinical management of AAA.

## Methods

### Resource of data and processing strategy

The expression patterns of mRNAs [GSE57691 [20] and GSE47472 [21]] in human tissue samples from AAA group and normal group were obtained from GEO database, which were filtered, merged, probe-annotated, normalised, and batched corrected using ‘‘sva’’ and ‘‘limma’’ R package [22] for further WCGNA and differential expression analysis. The Single-cell RNA-sequencing dataset GSE166676 [23] for AAA was downloaded for cell clustering and expression profile analyses. The microarray dataset GSE57691 was used to identify the key genes for the differential diagnosis of AAA and AS. All database information descriptions are presented in Supplementary Table 4.

### Assessment of immune cell infiltration

Single-sample GSEA analysis (ssGSEA) was performed using the ‘‘GSVA’’ R package [24] to reveal 28 types infiltrating immune cells between AAA and no-AAA and visualised using the "pheatmap" R package. The gene expression matrix was analysed using the CIBERSORT algorithm [25] to compare the infiltration differences of 22 immune cells in the AAA group and the non-AAA group. All samples are filtered according to the p-value ≤ 0.05 and then the immune cell infiltration matrix is output. The visual analysis of correlations of 22 types of infiltrating immune cells was performed using the ‘‘corrplot’’ R package [26].

### Identification of macrophage cell marker genes by scRNA-seq

The scRNA-seq data from the GSE166676 database were filtered according to the following three measures to obtain high-quality scRNA-seq data: retaining genes expressed in at least five single cells, eliminating less than 100 genes, and removing cells with mitochondrial genes exceeding 5%. We first perform the ‘‘NormalizeData’’ function to normalise scRNA-seq data by using the ‘‘Seurat’’ R package [27]. We converted the normalised data into a Seurat object and performed the ‘‘FindVariableFeatures’’ function to screen the top 2000 highly variable genes. Subsequently, we performed PCA analysis to reduce the scRNA-seq data dimension of the top 2000 genes by using the ‘‘RunPCA’’ function of the ‘‘Seurat’’ R package. Then batch effects were removed using the Harmony algorithm [28], and the datasets were integrated with the “merge” function from the “Seurat” R package. We performed PCA analysis to identify significant PCs and screened the top 15 PCs based on the variance ratio for cell clustering analysis. Cell clustering was performed on a shared nearest-neighbour graph with the Louvain algorithm. We use the ‘‘FindAllMarkers’’ function in the ‘‘Seurat’’ R package to calculate the differentially expressed genes (DEGs) for each cell cluster. We identify the marker genes of each cluster cell according to the following criteria: |LogFC|> 1 and *t* adjusted *p*-value ≤ 0.01. Finally, we annotated the clusters by referring to the human primary cell atlas data [29].

### Analysis of the GO and KEGG pathway of macrophage-related genes

We used the ‘‘clusterprofiler ’’R package [30] to perform Gene Ontology (GO) and Kyoto Encyclopedia of Genes and Genome (KEGG) analysis to reveal the biological functions and signal pathways involved in the above macrophage marker genes. Statistical significance was set at *p* < 0.05 for enriched functions and signalling pathways.

### Construction of co-expressing gene module for AAA

The distinct infiltrating immune cells identified by the CIBERSORT algorithm were further analysed using the WGCNA algorithm. During module construction, the power value was screened to guarantee the high-scale independence and average connectivity of the module. Module-trait relationships were analysed between modules and differential infiltrating immune cell types using Pearson’s correlation test, and a *p*-value < 0.05 was considered significant. Focusing on macrophages, we drew scatter plots of gene significance and module membership to define hub genes.

### Screening and validation of diagnostic biomarkers for AAA

Three methods were used to screen diagnostic biomarkers for AAA: single-cell differential gene analysis, identification of differentially expressed genes between AAA and non-AAA groups, and WCGNA. Genes at the intersection were selected as candidate diagnostic biomarkers and were further verified using the GSE57691 and GSE47472 datasets. Diagnostic efficiency was evaluated by plotting ROC curves [31].

### Development of the AAA mice model

Animal experiments were performed with the approval of the Experimental Animal Ethics Committee of the First Affiliated Hospital of Harbin Medical University. Here, 10 8 week-old male C57BL/6 mice were purchased from Vital River Laboratory Animal Technology Corporation (Beijing, China). The mouse abdominal aortic aneurysm model refers to previous literature [32]. In summary, the mice were provided with 0.2% 3-aminopropionitrile fumarate salt drinking water 2 days before surgical treatment until the end of this study. After the mice were anaesthetised, the lower 3/4 infrarenal abdominal aorta was exposed and separated from the vena cava. The exposed adventitia of the abdominal aorta was soaked in 20 µl elastase for 20 min. The abdominal cavity was flushed with normal saline three times and the abdomen was sutured and sterilised. At 28 days postoperatively, the mice were sacrificed, and the abdominal aorta was dissected for further use.

### Establishing and assessing the risk model of AAA

A nomogram model for predicting AAA was established using six hub genes (IL-1B, CXCL1, SOCS3, SLC2A3, G0S2, and CCL3) through multivariable logistic regression analysis. Calibration and decision curve analysis (DCA) were used to assess the performance of the nomogram [33]. Furthermore, clinical impact curve (CIC) analysis was performed to evaluate the clinical effectiveness of the nomogram using the ‘‘RMS’’ R package [34, 35].

### Patients with AAA and healthy individuals

We collected 20 patients with abdominal aortic aneurysm (AAA group) and 15 healthy individuals (non-AAA group) admitted to the First Affiliated Hospital of Harbin Medical University from September 2016 to July 2023. The diagnosis of AAA refers to the following standards [36]. This study was approved by the Ethics Committee of the First Affiliated Hospital of Harbin Medical University. All participants have signed informed consent forms. The clinical information of the participants is presented in Supplementary Table 7.

### Mining potential therapeutic agents for AAA

The connectivity Map (CMap) is a gene expression profiling database that connects small molecules, genes, and diseases [37]. We queried the CMap database to identify potential therapeutic agents for AAA. The similarity enrichment score was determined, ranging from − 1 to + 1. A positive connectivity score reflected the level of similarity, whereas a negative value denoted an inverted similarity. We then obtained comprehensive information on the small molecules from the Clue command and confirmed their 3D structure using PubChem (https://pubchem.ncbi.nlm.nih.gov/).

### Construct a potential regulatory network for the above six hub genes

Transcriptional regulatory relationships revealed by sentence-based text mining (TRRUST) (https://www.grnpedia.org/trrust/) is a database that identifies translational regulatory relationships of transcription factors (TFs) via literature mining [38]. We uploaded six hub genes to the database and selected TFs based on their regulatory connection relationships with the key genes. Next, a regulatory network was established and visualised using Cytoscape according to the relationship between TFs and six hub genes [39]. Furthermore, we predicted miRNAs and lncRNAs targeting the above six hub genes using the miRwalk and miRanda databases and constructed a competitive endogenous RNA (ceRNA) network.

### Screening and verification of diagnostic biomarkers between AAA and AS

Three algorithms were adopted to screen and verify the diagnostic biomarkers between AAA and AS: least absolute shrinkage selection (LASSO), Support Vector Machine (SVM), and Random Forest (RF) [40–42]. We performed RF algorithm using ‘‘randomForest ’’ R package and LASSO regression analysis using ‘‘glmnet’’ R package [43], with low lamda. The SVM classifier was established using ‘‘e1071’’ R package and RFE algorithm was utilised to select the optimal genes. Overlapping genes from the above three machine learning methods were then selected. The diagnostic biomarkers were validated using dataset GSE57691 and the diagnostic efficacy was assessed by ROC curves.

### Gene set variation analysis (GSVA)

GSVA is a nonparametric, unsupervised method for estimating variations in key gene sets. We utilised the "GSVA" R package [24] to determine the differential KEGG pathways in high and low expressed candidate genes with the data downloaded from the Molecular Signatures Database.

### Quantitative real-time PCR (RT-qPCR)

Total RNA was extracted from the samples using TRIzol Reagent (Invitrogen, USA) following the manufacturer’s instructions, and the purity of the collected RNA was determined using a NanoDrop 2000 spectrometer (Thermo Fisher Scientific, MA, USA). Next, the total RNA was reverse-transcribed into cDNA using the PrimeScript kit (Takara). qPCR was performed using the SYBR Green reagent kit (Takara) on an ABI 7500 PCR system (Applied Biosystems). The primer sequences are listed in Supplemental Tables 2, 3. The relative expression level of target mRNA was normalised with endogenous control GAPDH and calculated using the 2-^∆∆Cq^ method. Statistical significance was set at *p* < 0.05.

### Statistical analysis

All statistical analyses and charts were generated using the R software (version 4.3) and Graphad Prism (version 8.03). Comparisons between two groups were performed using the t-test, and comparisons between multiple groups were performed using the Wilcoxon signed-rank test. Statistical significance was set at p < 0.05.

## Results

### Characteristics of immune microenvironment in AAA

**Fig. 1:**
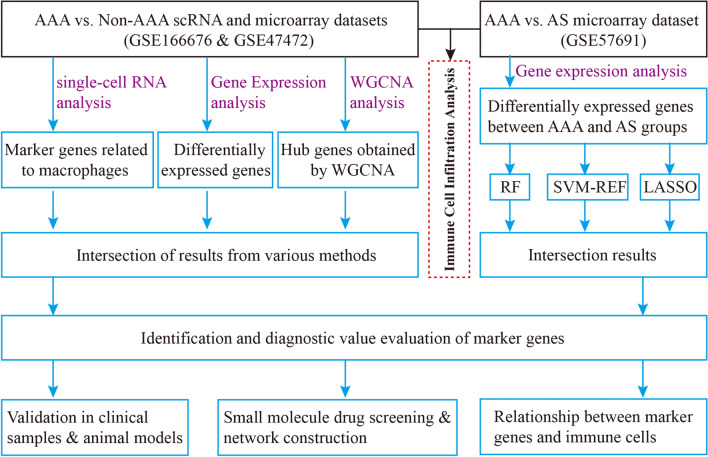
Flow chart of the research design

**Fig. 2:**
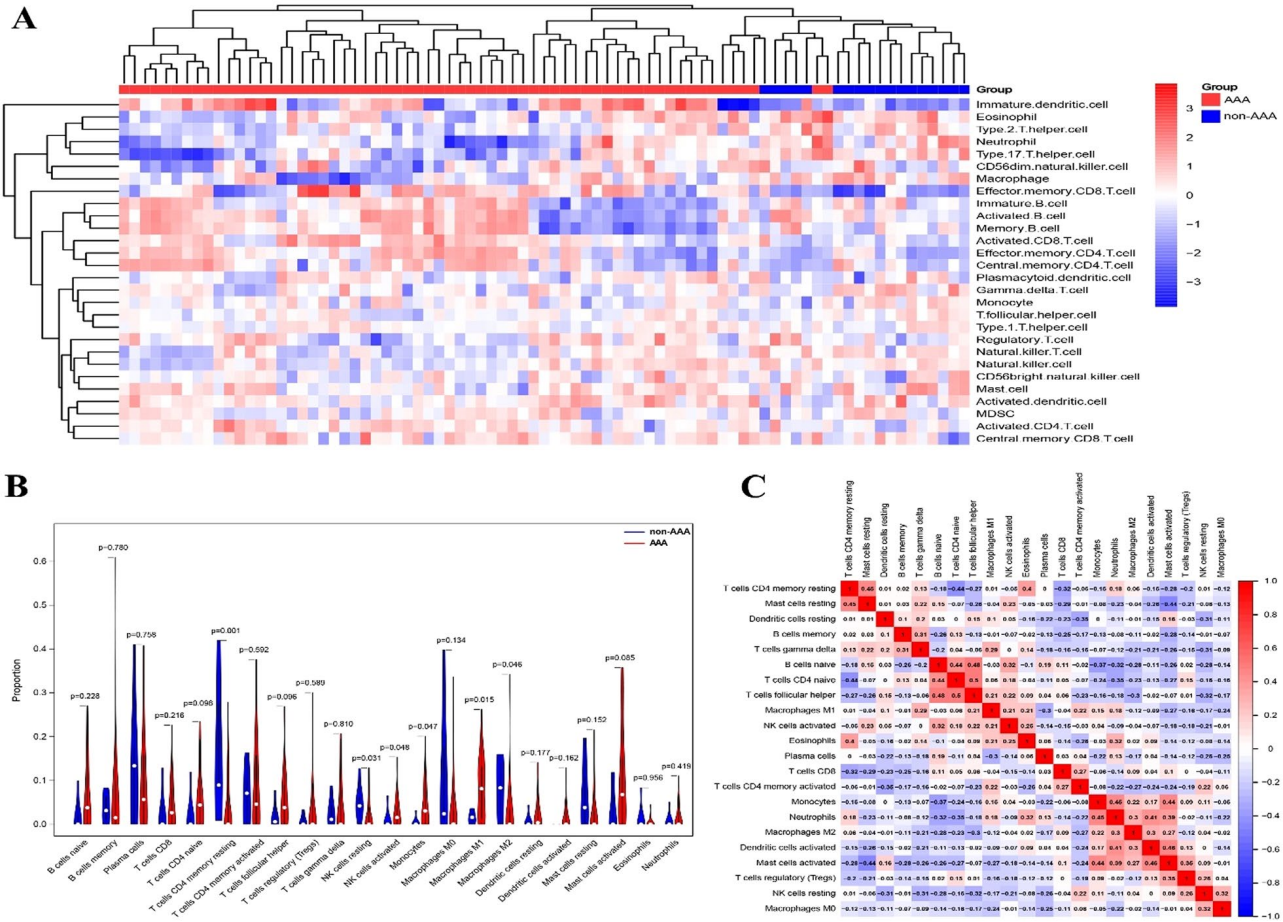
Landscape of immune cell infiltration in AAA and non-AAA tissues. **A** Clusters of infiltrating immune cells in AAA and non-AAA tissues. Rows represent infiltrating immune cells, and columns represent samples. **B** Differential immune cell infiltration in AAA and non-AAA tissues was analysed using the CIBRTSORT algorithm, and *p* < 0.05 was considered statistically significant. **C** Correlation heatmap of 22 types of immune cells. The correlation coefficient was displayed by colour, with darker colour showing stronger correlations. Red: Positive correlation; Blue: negative correlation

To provide a comprehensive overview of our experiments, a flowchart of the study is presented in Fig. 1. Expression profiling data from GSE57691 and GSE47472 were filtered, merged, normalised, and batch corrected. Next, 28 immune cell types in each sample were analysed using ssGSEA. The landscape of immune cell infiltration in AAA and non-AAA tissues is shown in Fig. 2A. CIBERSORT was used to calculate the abundance of immune infiltrates. AAA tissue contained a lower proportion of resting CD4 + memory T cells, natural killer (NK) cells, and macrophages M2, while the proportion of activated NK cells, monocytes, and macrophages M1 was relatively high (Fig. 2B). The correlation heatmap of the investigated immune cells showed that resting mast cells positively correlated with resting CD4 + memory T cells. Naïve CD4 T and B cells, eosinophils and T cells, resting CD4 memory T cells, neutrophils and monocytes, activated dendritic cells and neutrophils, regulatory T cells (Tregs), and activated mast cells were positively correlated. Follicular helper T cells were positively correlated with activated NK cells, and naïve B and CD4 T cells. Activated mast cells were positively correlated with monocytes, neutrophils, macrophages M2, and activated dendritic cells (aDCs). A negative correlation was observed between dendritic cell activation and macrophage M1, CD4 naïve T cells and CD4 memory resting T cells, activated mast cells and resting mast cells, monocytes and naïve B cells, and monocytes and T cells CD4 naïve (Fig. 2C). These results indicated that there are big differences in immune cell infiltration and expression patterns between AAA and non-AAA samples, which might imply new biomarkers and therapeutic targets yet to discover.

### Identification of macrophage marker gene expression profiles in AAA using scRNA-seq

**Fig. 3:**
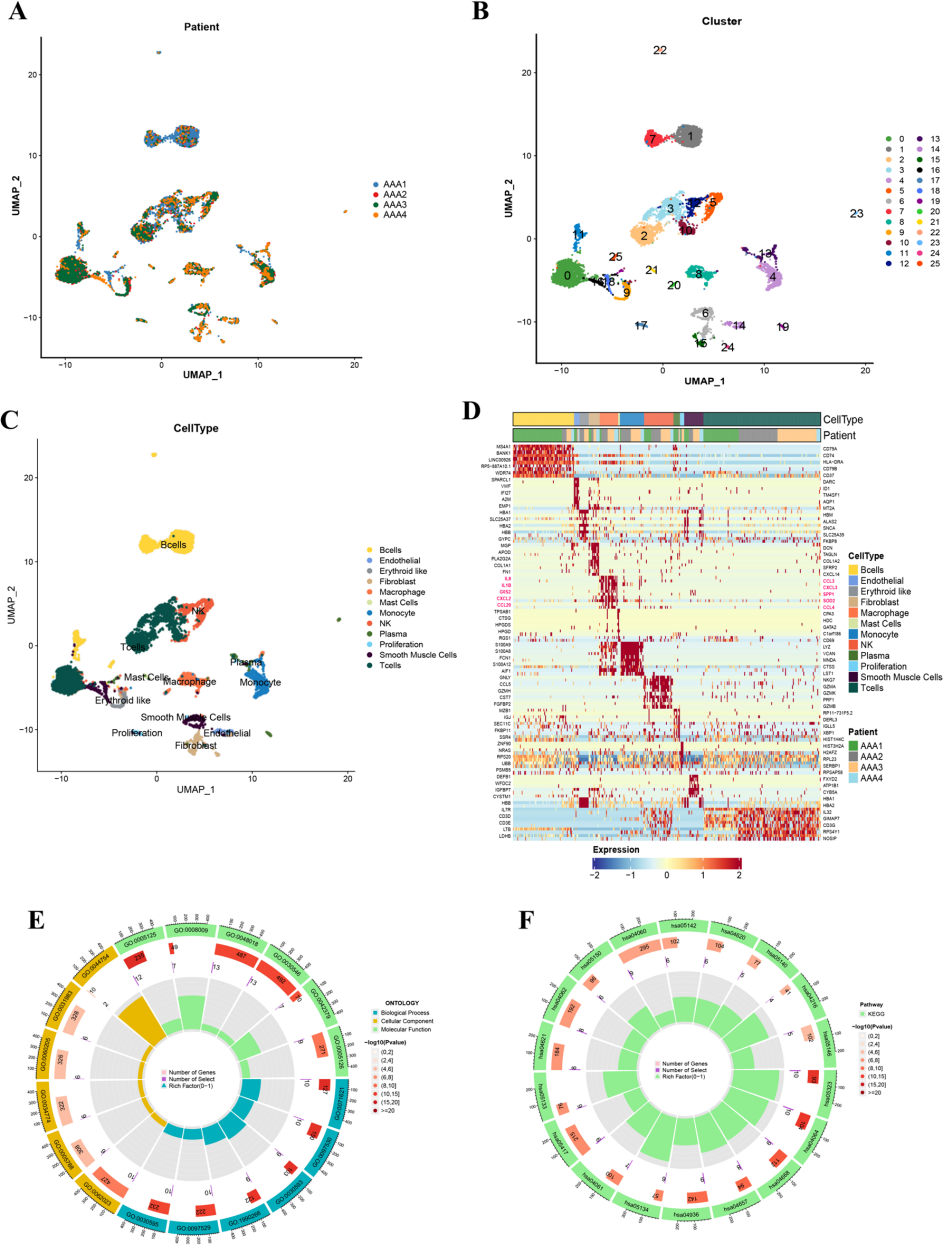
Single-cell sequencing and biological function analysis. **A** UMAP showing a clear separation of cells in 4 AAA tissues. **B** Cell clustering at 0.5 resolution. **C** All 26 cell types were labelled according to the composition of marker genes. **D** Heatmap of differentially expressed MRGs in 12 cell types grouped by patients, maximum top 50 genes showed per cell cluster. *p *< 0.001 and |log2FC|> 1 were identified as significant DEGs. **E** GO term analysis of top 50 MRGs. **F** KEGG enrichment analysis of top 50 MRGs

Single-cell RNA sequencing data from four AAA patients (GSE166676) were extracted for data quality control, integration, and cell cluster identification (Fig. 3A). We used the top 2000 variable genes for principal component analysis (PCA) to reduce dimensionality and identified 26 cell clusters (Fig. 3B). We annotated the identities of clustered cells using a reference dataset of human primary cell atlases. As shown in Fig. 3C, the major immune cell types were B cells (clusters 1, 7, 11, and 25), T cells (clusters 0, 2, 3, and 10), NK cells (clusters 5 and 12), monocytes (cluster 4), macrophages (cluster 8), and mast cells (cluster 21). As the above analysis revealed differences in macrophages between AAA and non-AAA tissues, we focused on macrophage clusters. We defined the differentially expressed genes between cluster8 and the other clusters as AAA-associated macrophage marker genes. The top 50 differentially expressed marker genes in macrophages and other immune cell types are shown in a gene expression heatmap (Fig. 3D) (Supplemental Table S1). The function of differentially expressed marker genes was examined by enriching the GO and KEGG databases. GO functional annotation of differentially expressed marker genes showed that 18 terms were enriched involving genes regulating proteins with cytokine activity (GO:0005125), chemokine activity (GO:0008009), receptor ligand activity (GO:0048018), signalling receptor activator activity (GO: 0030546), chemokine and cytokine receptor binding (GO:0042379, GO:0005126), granulocyte chemotaxis and migration (GO:0071621, GO:0097530), neutrophil chemotaxis and migration (GO:0030593, GO:1990266), myeloid leukocyte migration and leukocyte chemotaxis (GO:0097529, GO:0030595), collagen-containing extracellular matrix (GO:0062023), endoplasmic reticulum lumen (GO:0005788), secretory granule lumen (GO:0034774), cytoplasmic vesicle lumen (GO:0060205), and vesicle lumen and autolysosome (GO:0031983, GO:0044754), which can be seen in Fig. 3E. In Fig. 3F, KEGG pathway enrichment analysis demonstrated that 18 pathways were enriched among the differentially expressed marker genes, mainly related to immune and inflammatory signalling pathways, such as NF-kappa B signalling pathway (has04064), TNF signalling pathway (hsa04668), IL-17 signalling pathway (hsa04657), NOD-like receptor signalling pathway (hsa04621), chemokine signalling pathway (hsa04062), cytokine-cytokine receptor interaction (hsa04060), and Toll-like receptor signalling pathway (hsa04620). Notably, lipid metabolism, atherosclerosis, and ferroptosis were enriched (hsa05417 and hsa04216). These results provide valuable insights into the immune cell infiltration and expression patterns in AAA, highlighting macrophages and their involvement in immune and inflammatory signalling pathways. Further validation and extension of the study to a larger cohort are needed to confirm the findings.

### WCGNA analysis and identification of macrophage relevant modules

**Fig. 4:**
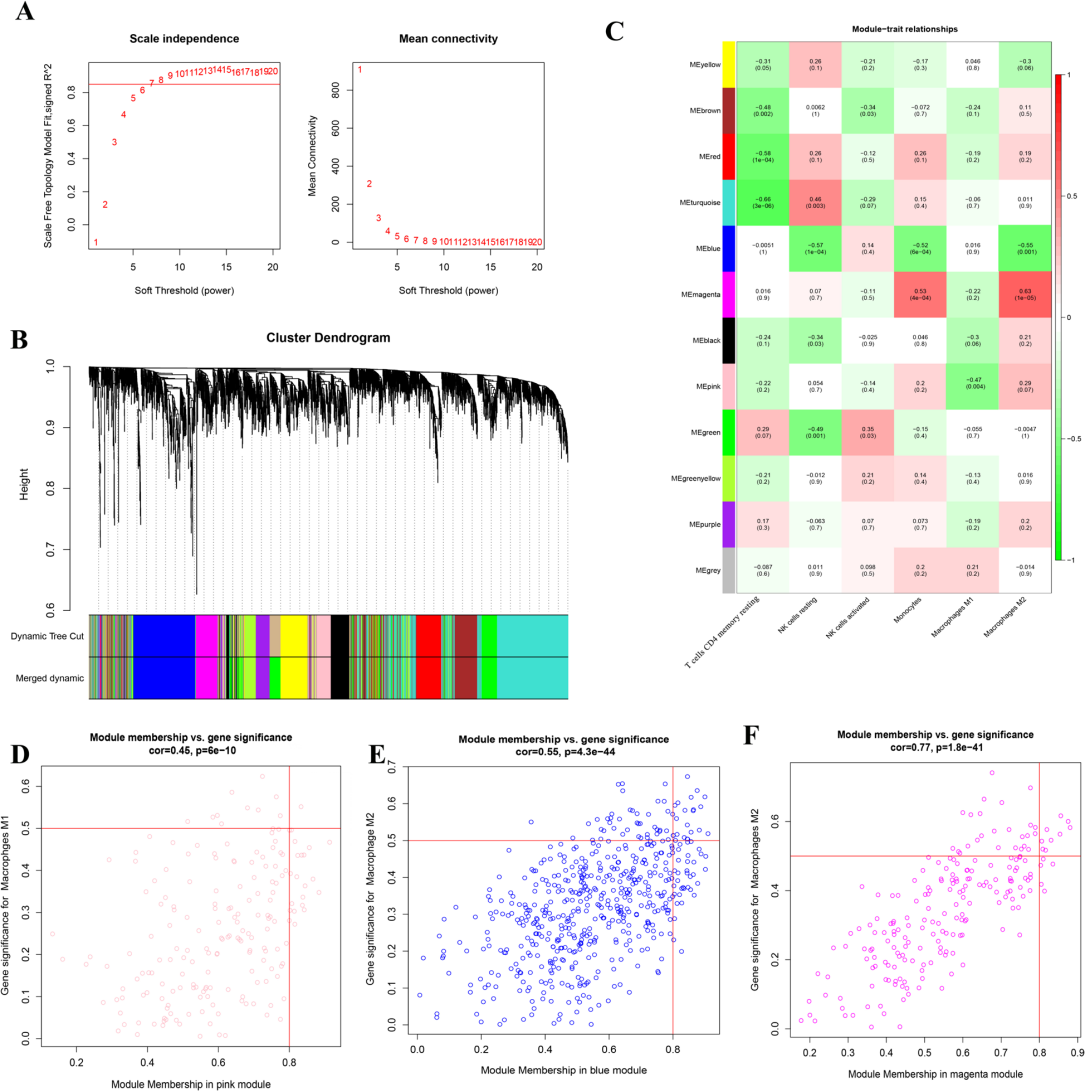
Weighted gene co-expression network analysis of six differentially infiltrating immune cells in AAA. **A** Effect of optimal soft threshold power value on the scale independence and mean connectivity of genes co-expression modules. **B** Clustering dendrogram of genes in six differentially infiltrating immune cells. **C** Correlation analysis of modules and six differentially infiltrating immune cells. Three macrophage-related modules were selected. **D**–**F** Scatter plot of the pink module, blue module, magenta module showing the relationship between module membership and gene significance

We previously examined the features of immune cell infiltration. Six types of immune cells that varied between the AAA and non-AAA groups were retained for subsequent WCGNA. After eliminating outlier samples, a scale-free network (the soft threshold power for matrix transformation was set to 7, in which *R*^2^ = 0.85) and a mean connectivity network were established (Fig. 4A). Different co-expression modules with different colours were constructed after hierarchical clustering and dynamic branch cutting (Fig. 4B). Six differentially infiltrating immune cells were linked to gene expression modules. As shown in Fig. 4C, the pink module (*r* = − 0.47, *p* = 0.004) was associated with M1 macrophages, whereas the blue (*r* = − 0.55, *p* = 0.001) and magenta modules (*r* = 0.63, *p* = 1e–05) were closely correlated with M2 macrophages. We constructed scatterplots of module membership versus gene significance for pink, blue, and magenta modules. They had positive correlations of 0.45, 0.48, and 0.77 with a significant *p*-value of < 0.001 (Fig. 4D-F). By performing WGCNA, we established a scale-free network model and identified specific gene expression modules associated with immune cells. These results provide new insights into the immune cell infiltration mechanisms of AAA and may serve as a foundation for future diagnostic and therapeutic strategies.

### Identification and validation of hub genes for AAA

**Fig. 5:**
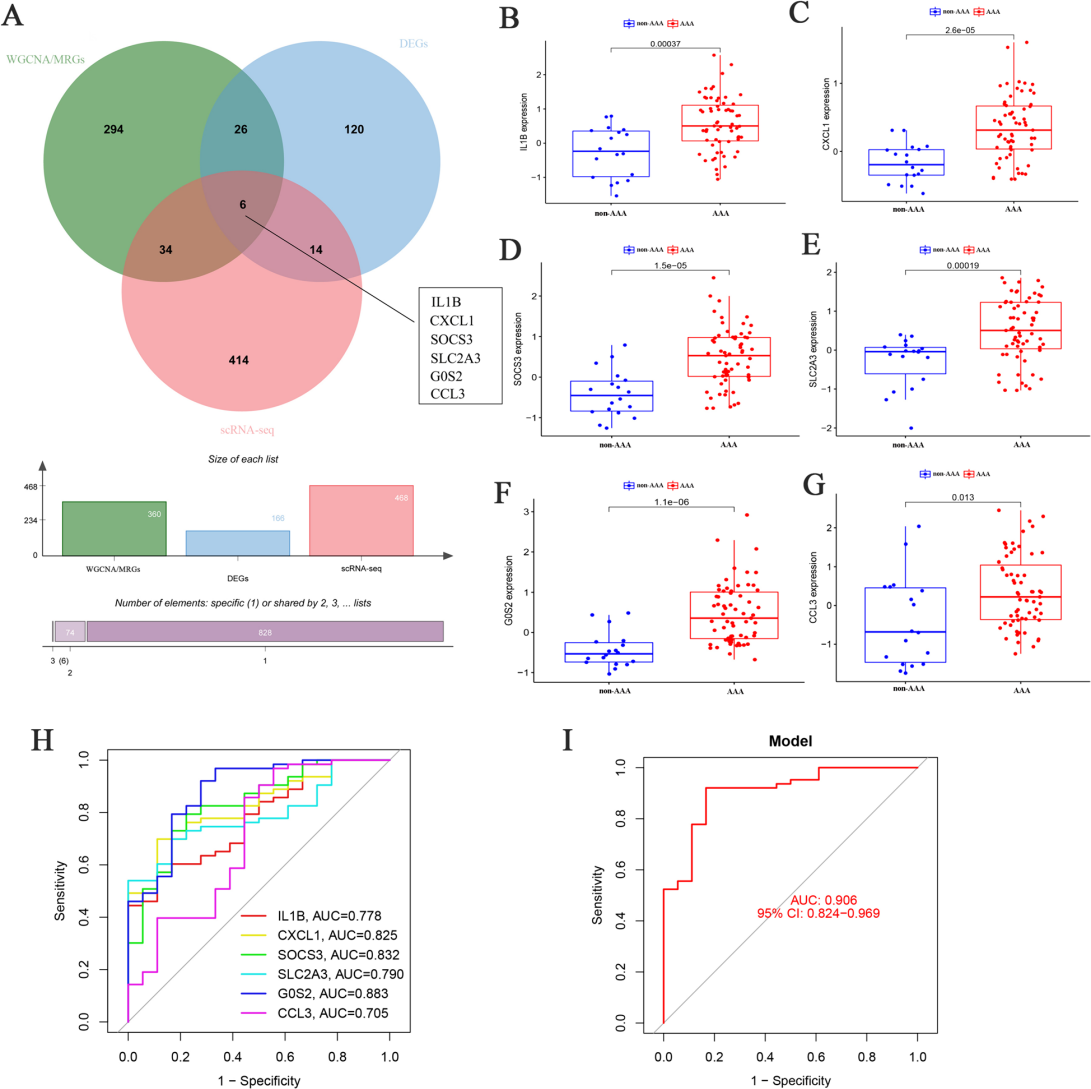
Identification and validation of key genes for AAA. **A** Six key genes are at the intersection based on Venn diagram, including IL-1B, CXCL1, SOCS3, SLC2A3, G0S2, and CCL3. **B**–**G** Validating the expression levels of six key genes in AAA and non-AAA patients using GSE57691 and GSE47472 database. **H** ROC curves showed the diagnostic value of six key genes for AAA. **I** ROC curve showed the combined diagnostic value of six key genes for AAA

By plotting a Venn diagram, six hub genes, IL-1B, CXCL1, SOCS3, SLC2A3, G0S2, and CCL3, were identified at the intersection of macrophage-related genes (MRGs), differentially expressed genes (DEGs) and scRNA-seq (Supplemental Table 5), indicating that these genes contributed to the development of AAA (Fig. 5A). We further validated the expression levels of the six hub genes using the GEO database (GSE47472 and 57,691) and found that all genes were highly expressed in AAA tissues compared to non-AAA tissues (Fig. 5B-G). Subsequently, ROC curve analysis was performed to assess the sensitivity and specificity of IL-1B, CXCL1, SOCS3, SLC2A3, G0S2, and CCL3 for the diagnosis of AAA. As exhibited in Fig. 5H-I, the area under the curve (AUC) values of IL-1B, CXCL1, SOCS3, SLC2A3, G0S2, and CCL3 were 0.778, 0.825, 0.832, 0.790, 0.883, and 0.705, respectively (Fig. 5H). Moreover, the combined diagnostic performance of the six hub genes was higher than that of a single gene, with an AUC value of 0.906 (Fig. 5I), indicating an excellent diagnostic efficacy for AAA.

### Verification of diagnostic biomarkers in mice and human samples

**Fig. 6:**
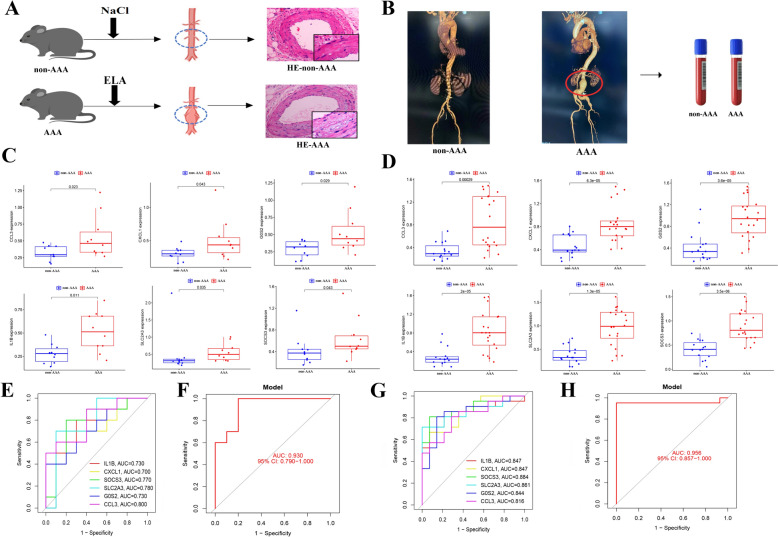
Verification of diagnostic biomarkers in mice and human serum. **A** Construction of AAA mice model using CaCl_2_ and verified by HE staining. **B** Clinical serum was collected from people with AAA. **C** Validating the expression levels of six key genes in mice tissues using qRT-PCR. **D** Validating the expression levels of six key genes in human serum using qRT-PCR. **E** ROC curves based on the expression of six key genes in mice tissues. **F** ROC curve showed the combined diagnostic value of six key genes based on mice tissues. **G** ROC curves based on the expression of six key genes human serum. **H** ROC curve showed the combined diagnostic value of six key genes for AAA based on human serum

To confirm the applicability of this diagnostic model, we validated its effectiveness of the diagnostic model in AAA mice and clinical samples. As shown in Fig. 6A–B, we established an AAA mouse model and screened patients with AAA using CT angiography (CTA). The expression levels of six hub genes in 20 mouse tissue samples (10 normal and 10 AAA samples) and 35 patient serum samples (15 non-AAA and 20 AAA patient samples) were detected using RT-qPCR. In contrast to non-AAA tissues, AAA tissues in mouse tissue showed higher expression levels of CCL3, CXCL1, G0S2, IL-1B, SLC2A3, SOCS3 (Fig. 6C). Consistent with this, the serum expression levels of the six hub genes were also higher in patients with AAA than in the control group (Fig. 6D). To further verify the diagnostic value of the six hub genes, ROC curves were constructed based on their expression levels in mouse tissues and human serum. The AUC values of IL-1B, CXCL1, SOCS3, SLC2A3, G0S2, and CCL3 were calculated to be 0.73, 0.70, 0.77, 0.78, 0.73, and 0.80 in mice tissues, and 0.847, 0.847, 0.884, 0.861, 0.844, and 0.816 in human serums, respectively (Fig. 6E, 6G). Combinations of the six hub genes showed excellent performance with AUC values of 0.930 and 0.983 for mouse tissues and human serum, respectively (Fig. 6F, 6H). These findings provide strong evidence for the potential use of these biomarkers in diagnosing AAA.

### Construction and assessment of the nomogram model of AAA

**Fig. 7:**
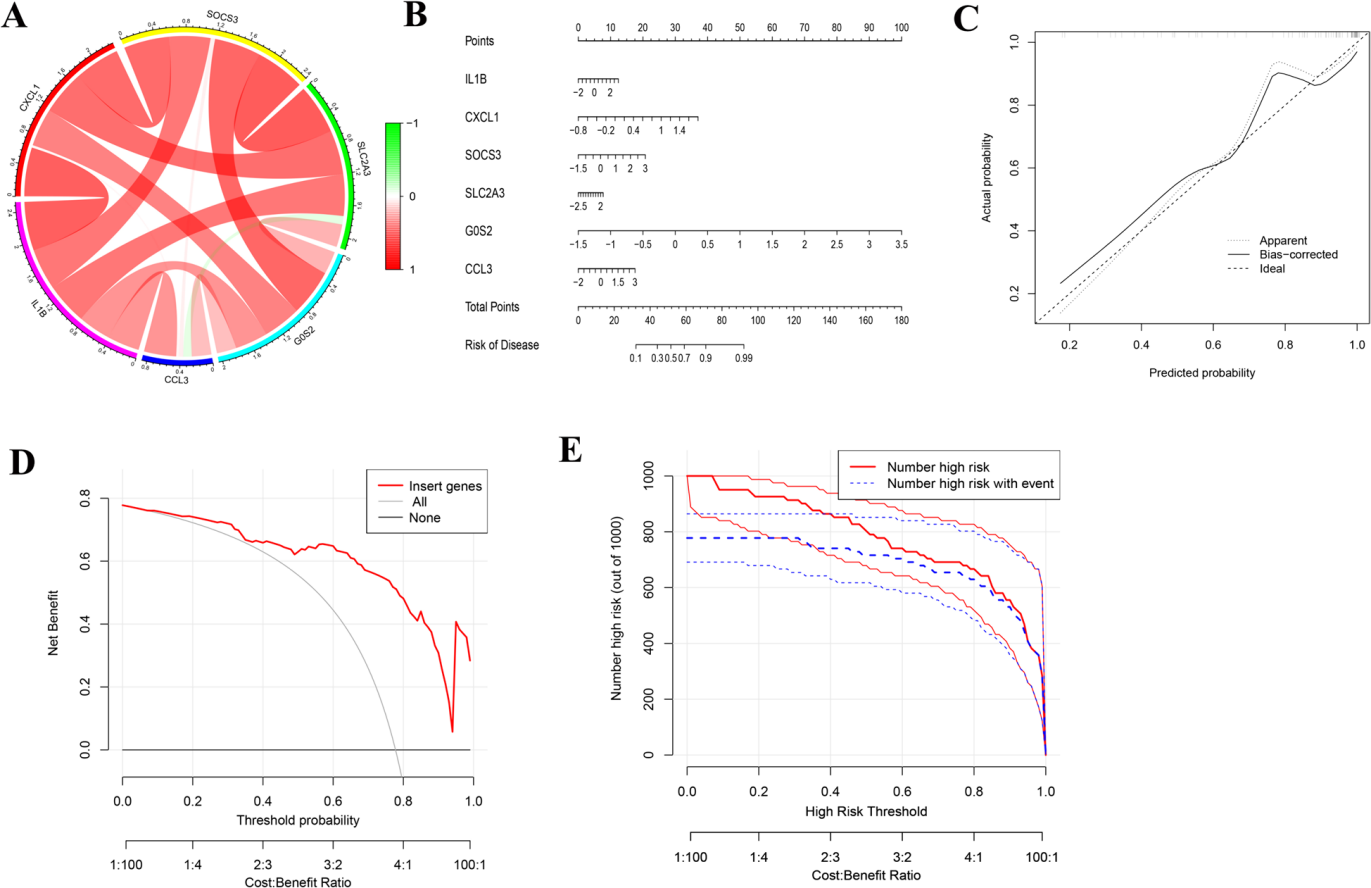
Risk score model for predicting AAA. **A** Chord diagram depicting the connections between six key genes. **B** Nomogram for prediction of AAA based on six key genes. **C** Calibration curves for the nomogram predicting AAA. The x-axis represents the predicted probability estimated by nomogram, and y-axis represents the actual probability of AAA. **D** Decision curve analysis for nomogram. The net benefit was plotted against the threshold probability and cost–benefit ratio of deciding to diagnose as AAA. **E** Clinical impact curve (CIC) of the nomogram. The red curve (number of high-risk individuals) indicates the number of people who are predicted as AAA by the nomogram at each threshold probability. The blue curve (number of high- risk individuals with event) is the number of actual AAA individuals at each threshold probability

We further revealed the correlation of the expression of the six hub genes in AAA using a chord diagram. As shown in Fig. 7A, IL-1B and G0S2 are highly correlated with other macrophage-related hub genes in AAA, with IL-1B and CXCL1 being the most correlated genes. We constructed a nomogram based on the characteristics of the six hub genes to establish a quantitative scoring model which could predict the prevalence of AAA (Fig. 7B). The calibration curve revealed that the prediction model was consistent with the actual probability (Fig. 7C). Moreover, Decision Curve Analysis (DCA) was applied to estimate the clinical utility of the nomogram model, and DCA showed that the predictive model could gain more net benefits, with a threshold probability ranging from 0.1 to 1.0 (Fig. 7D), suggesting a good potential for clinical application. The clinical impact curve (CIC) is a graph to consider the potential harm to patient interests caused by clinical misdiagnosis and missed diagnosis. In Fig. 7E, the CIC plot showed the number of high-risk patients predicted by the nomogram and the actual number of high-risk patients with events for each risk threshold. The predicted number of high-risk patients was close to the actual number of high-risk patients with events when the high-risk threshold probability was > 0.5.

### Ming potential therapeutic agents for AAA and construction of the TF gene, lncRNA–miRNA–mRNA network in AAA

**Fig. 8:**
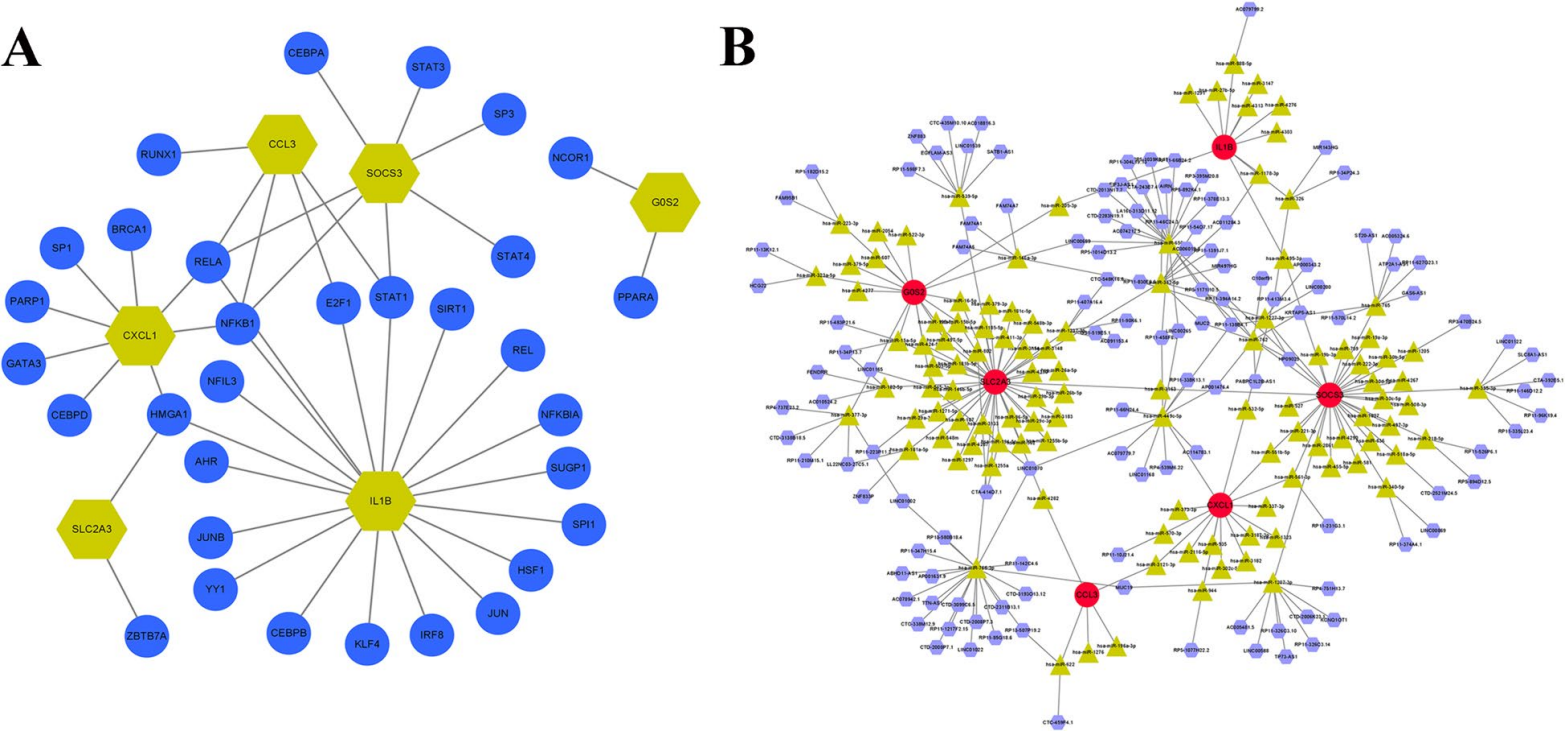
Potential therapeutic mediations and gene networks for targeted therapies. **A** Transcriptional regulatory network of six key genes and transcriptional factors. **B** Transcriptional regulatory network of six key genes, miRNAs, and lncRNAs

We identified 34 potential small-molecule drugs targeting AAA using the CMap database. As shown in Table 1, we identified eight small-molecule drugs: prostratin, calmidazolium, YM-155, LDN-193189, CD-437, SCH-79797, JTC-801, and penfluridol (Table 1). We subsequently revealed the three-dimensional structures of the above eight small-molecule drugs (Supplemental Fig. S1A–H) through online websites (https://clue.io/query) and speculated that they may affect specific gene changes to alleviate AAA. We further explored the regulatory mechanisms of these six hub genes. The TFs of six hub genes were identified, and a TF gene regulatory network was constructed, including 43 interaction pairs among the six hub genes and 32 TFs (Fig. 8A). The same gene is regulated by various varieties of TFs. For example, CXCL1 is regulated by BRCA1, SP1, PARP1, GATA3, CEBPD, HMGA1, NFκB1, and RELA. G0S2 is regulated by NCOR1 and PPARA. The interactions between lncRNAs, miRNAs, and mRNAs should be considered. Considering that lncRNAs can regulate the expression of their corresponding target genes through miRNAs, we constructed a lncRNA–miRNA–mRNA regulatory network targeting the above hub genes (Fig. 8B) to reveal the potential molecular mechanisms involved in the occurrence and development of AAA. The ceRNA network contained 6 mRNA nodes, 110 miRNA nodes, and 126 lncRNA nodes.

**Table 1:** The top eight small-molecule drugs identified by DEGs

Rank	CMap name	Score	Description
1	Prostratin	99.93	Prostratin PKC activator
2	Calmidazolium	99.89	Calcium channel blocker
3	YM-155	99.75	Survivin inhibitor
4	LDN-193189	99.68	Serine/threonine kinase inhibitor
5	CD-437	99.51	Retinoid receptor agonist
6	SCH-79797	99.12	Proteasome inhibitor
7	JTC-801	98.93	Opioid receptor antagonist
8	Penfluridol	98.27	T-type calcium channel blocker

### Identification and validation of key diagnostic biomarkers of atherosclerosis and abdominal aortic aneurysm

**Fig. 9:**
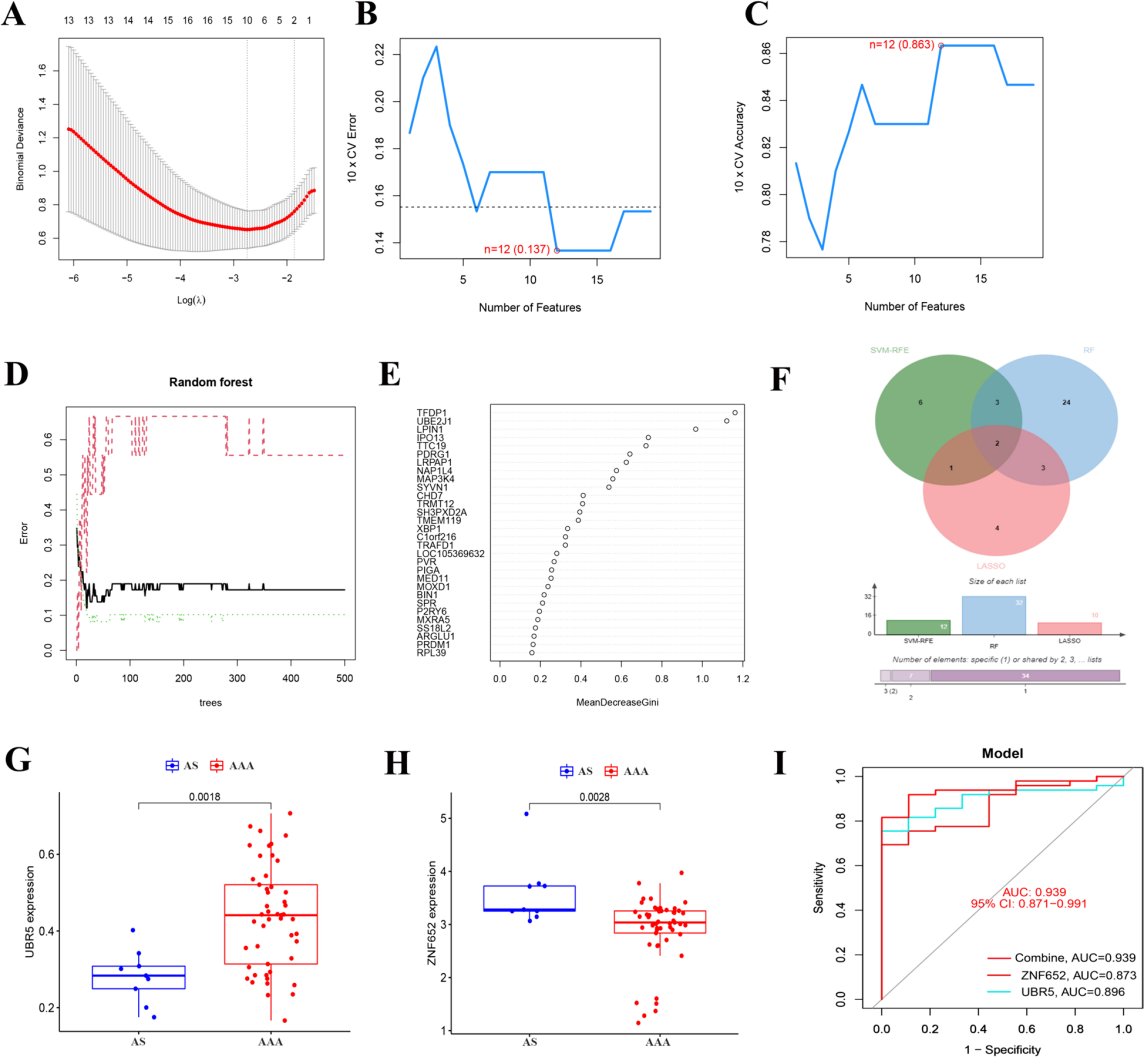
Identification and validation of differential diagnostic biomarkers of atherosclerosis and abdominal aortic aneurysm. **A** LASSO regression analysis. **B**–**C** SVM-RFE algorithm. **D**–**E** RF algorithm. **F** Venn plot exhibiting the critical biomarkers among LASSO, SVM-RFE, and RF. **G**–**H** Validation of two differential diagnostic biomarkers in Atherosclerosis (AS) and abdominal aortic aneurysm (AAA). **I** ROC curves showed the diagnostic capacity of ZNF652, UBR5, and a combination of two biomarkers for distinguishing AS and AAA

AS and AAA are the two most common vascular diseases. Atherosclerosis is a significant independent risk factor for abdominal aortic aneurysms for several years [44]. To identify the most relevant genes for distinguishing between AAA and AS, three machine learning methods were applied: LASSO logistic regression (Fig. 9A), SVM-RFE (Fig. 9B–C), and RF (Fig. 9D–E). As shown in the Venn diagram, 10, 12, and 32 most critical genes were screened using LASSO, SVM-RFE, and RF, respectively (Fig. 9F). Two overlapping genes (ZNF652 and UBR5) were selected as critical diagnostic biomarkers to distinguish between AAA and AS. We confirmed the expression levels of these two critical diagnostic biomarkers using the GEO database (GSE57691). The expression level of UBR5 was significantly higher in AAA compared to AS (Wilcoxon test, *p-*value = 0.0018), while the expression level of ZNF652 was notably lower in AAA than in AS (Wilcoxon test, *p*-value = 0.0028), as illustrated in Fig. 9G–H. The ROC curve showed that ZNF652 and UBR5 could discriminate between AAA and As, with AUC of 0.873 and 0.896, respectively. Additionally, the combination of the two biomarkers showed better diagnostic efficacy, with an AUC of 0.939 (Fig. 9I).

### Immunological characteristics of AAA and AS

**Fig. 10:**
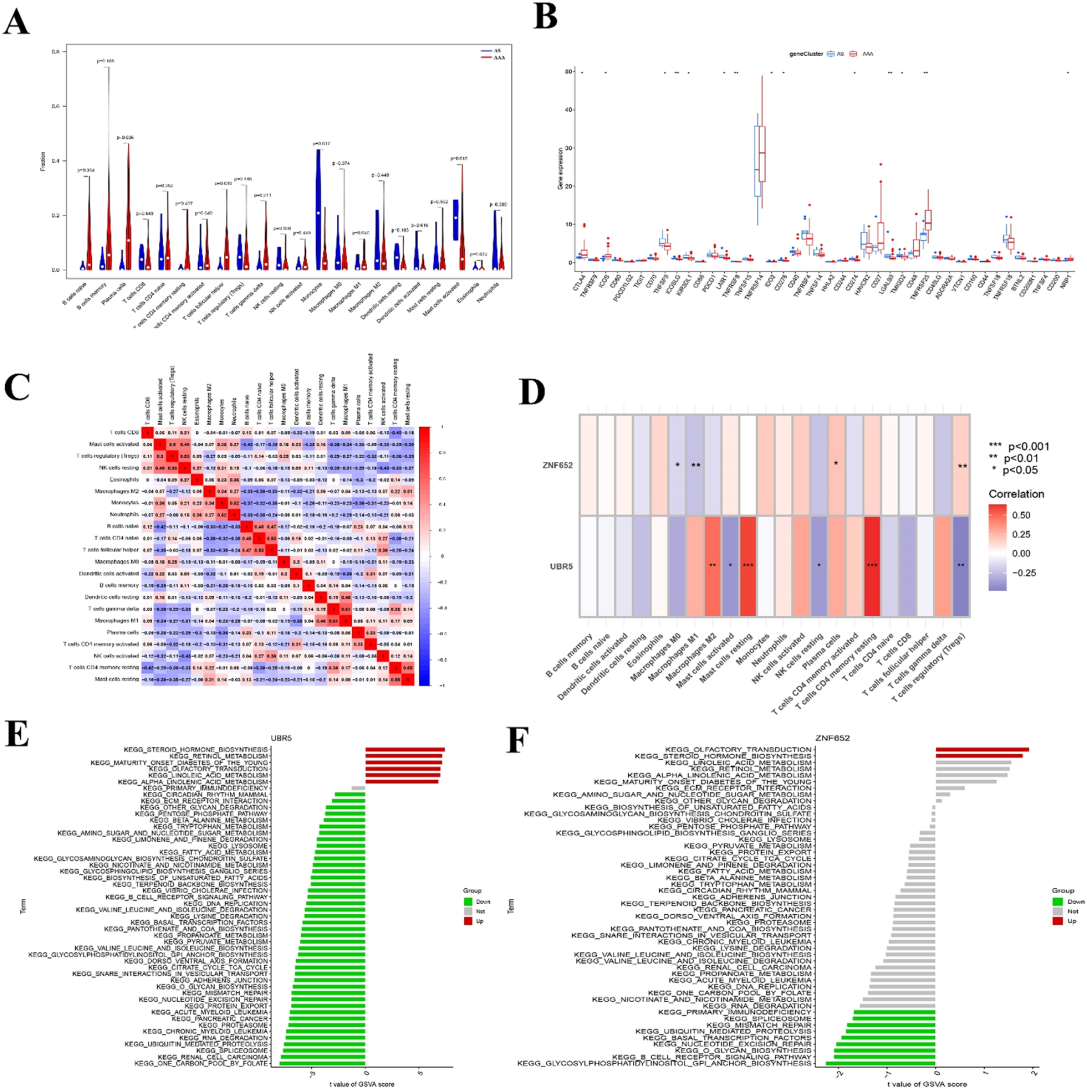
Immunological characteristics of AAA and AS. **A** Violin plot showing the difference in immune cell infiltration between AAA and As. **B** Box plot showed 42 Immune-related genes expressed in AAA and As. **C** Correlation heatmap of 22 different types of immune cells in AS. **D** Heatmap of the correlation between two biomarkers and 22 different types of immune cells. **E** KEGG enrichment analysis of UBR5 downregulated and upregulated group. **F** KEGG enrichment analysis of ZNF652 downregulated and upregulated group

CIBERSORT was used to reveal immune cell infiltration and characterise the immune properties of AAA and As. In Fig. 10A, the violin plot showed that plasma cells, follicular helper T cells, and macrophages M1 were higher in AAA tissues, whereas activated monocytes and mast cells were higher in AS tissues. A total of 42 immune checkpoint genes were analysed in both AAA and AS patients. CTLA4, ICOS, TNFRSF25, and NRP had significantly higher expression levels in patients with AAA than in those with AS, whereas TNFSF9, ICOSLG, KIR3DL1, LAIR1, TNFRSF8, IDO2, CD276, CD274, LGALS9, and TMIGD2 were distinctly lower in patients with AAA than in those with AS (Fig. 10B). A correlation heatmap of the 22 different types of immune cells is shown in Fig. 10C. Furthermore, correlation analysis was conducted to investigate the relationship between diagnostic biomarkers and immune cells. As shown in Fig. 10D, ZNF652 had a significant negative correlation with M0 (correlation = − 0.16, *p* < 0.05) and M1 (correlation = -0.23, *p* < 0.01) macrophages, and a significant positive correlation with plasma cells (correlation = 0.21, *p* < 0.05) and Tregs (correlation = 0.17, *p* < 0.01). UBR5 had a strong positive correlation with macrophages M2 (correlation = 0.48, *p* < 0.01), resting mast cells (correlation = 0.59, *p* < 0.001) and CD4 memory T cells (correlation = 0.64, *p *< 0.001), and a negative relationship with activated mast cells, resting NK cells (correlation = − 0.39, *p* < 0.05), and Tregs (correlation = − 0.48, *p* < 0.01). We further evaluated the expression levels of hallmark pathways between gene sets with high and low expression levels of UBR5 and ZNF652. GSVA results showed that compared with the low UBR5 expression group, pathways including steroid hormone biosynthesis, retinol metabolism, onset diabetes of the young, olfactory transduction, linoleic acid metabolism, and alpha-linoleic acid metabolism were upregulated in the group with high UBR5 expression (Fig. 10E). In the ZNF652 low-expression group, primary immunodeficiency, spliceosome, mismatch repair, ubiquitin-mediated proteolysis, basal transcription factors, nucleotide excision repair, O-glycan biosynthesis, B cell receptor signalling pathway, and glycosylphosphatidylinositol GPI anchor biosynthesis pathway were downregulated (Fig. 10F). These dysregulated pathways may also affect the development of AAA. Overall, these results provide valuable insights into the immunological characteristics of AAA and how they differ from AS, which might shade light on new therapeutic targets yet to discover.

## Discussion

AAA is a vascular disease characterised by a gradually enlarging aorta with structural destruction. It is insidious at an early stage, but can be lethal once ruptured. Unfortunately, effective nonsurgical interventions are lacking. It is necessary to elucidate the molecular mechanisms underlying AAA development, identify diagnostic biomarkers, and identify novel therapeutic agents for AAA.

In this study, we assessed immune cell infiltration in AAA tissues using the CIBERSORT algorithm. Significantly different infiltration percentages of resting CD4 memory T cells, resting NK cells, activated monocytes, and macrophages M1 and M2 were found between AAA and non-AAA tissues. We then conducted dimension-reduction clustering and identified the top 50 DEGs between macrophages and other immune cells based on single-cell sequencing data. Functional and pathway enrichment of the top50 genes was constructed, mainly related to immune and inflammatory biological processes. Next, WCGNA was performed to identify the modules of co-expressed genes that correlated with the six differentially infiltrated immune cells. Subsequently, six hub genes were obtained by intersecting the genes mined by single-cell sequencing analysis and WCGNA with the DEGs. These included IL-1B, CXCL1, SOCS3, SLC2A3, G0S2, and CCL3. The expression levels of the six hub genes were higher in the AAA group than in the non-AAA group, which was verified in both AAA mice and peripheral blood samples. ROC analysis was performed, and the AUC for their combined usage was 0.906, suggesting that they have potential clinical diagnostic value.

IL-1B, a multifunctional cytokine secreted mainly by activated macrophages and monocytes, plays an important role in the regulation of local and systemic inflammatory responses. Patients with AAA had higher serum IL-1B levels, which was particularly associated with rs35829419 polymorphism of the NLRP3 common allele [45]. CXCL1 is primarily expressed in neutrophils, macrophages, and epithelial cells and is a pro-inflammatory chemoattractant that plays a critical role in modulating the infiltration of neutrophils, macrophages, and monocytes [46]. The expression of CXCL1 in the vascular outer membrane triggers the recruitment and activation of neutrophils, leading to inflammation of the outer membrane and aortic dilation and rupture [47]. SOCS3 is a feedback inhibitor of the JAK/STAT signalling pathway that can inhibit STAT3 phosphorylation and regulate cytokine and hormone signalling and inflammatory responses [48]. In atherosclerosis, platelets induce SOCS3 expression in macrophages, causing them to differentiate into inflammatory phenotypes and secrete inflammatory cytokines, thereby promoting plaque formation [49]. SLC2A3 is a member of the solute carrier family, which functions as a transmembrane transporter with glucose transmembrane transport activity [50]. A retrospective clinical study on coronary atherosclerosis demonstrated that G0S2 expression in peripheral blood leukocytes is an independent risk factor for AMI in patients with stable CAD [51]. In addition, the overexpression of G0S2 can promote the transition of M2 macrophages to M1 macrophages, exacerbating inflammation progression in the kidney and accelerating renal fibrosis [52]. In a recently published study, G0S2 was highly correlated with AAA [53]. CCL3, also named MIP-1α, is produced and secreted by activated macrophages to attract other pro-inflammatory cells [54]. A recent study reported that CCL3 expression levels were elevated in CaCl_2_-induced AAA; however, the lack of CCL3 aggravated CaCl_2_-induced AAA formation by augmenting macrophage recruitment and macrophage-derived MMP-9 expression, suggesting its protective function [3]. We subsequently constructed a TF gene regulatory network, in which the transcription factor NFKB1 can regulate the expression of CXCL1, IL-1B, and SOCS3 mRNAs, suggesting that NFKB1 may play an important role in AAA disease. A recent study found that abnormal expression of NFKB1 in vascular adipose tissue can lead to immune response disorders and induce AAA occurrence [55]. In recent years, non-coding RNAs, especially lncRNAs and miRNAs, have been considered as gene expression regulators. A prior study reported that miR-146a-5P could inhibit macrophage pyroptosis and alleviate AAA [56]. Other studies reported that lncRNA FGD-AS1 could promote AAA occurrence by targeting miR-195-5P in vascular smooth muscle cells [57]. We have constructed a potential ceRNA regulatory network targeting six genes, providing insights into the potential mechanisms and targeted therapies of AAA. For anti-inflammatory genes, we can use corresponding lncRNA preparations to promote their expression. For pro-inflammatory genes, such as IL-1B, we can use corresponding miRNA preparations or IL-1B inhibitors, such as lionassep with diacerein, to inhibit the inflammatory response [58].

Atherosclerosis is a known cause of AAA, and there are many similarities between these two diseases. To identify markers that could distinguish between the two diseases, we applied three machine learning methods: LASSO logistic regression, SVM-RFE, and RF. Two genes, ZNF652 and UBR5, were screened and showed good diagnostic efficacy. There are some differences in the underlying molecular mechanisms of these two diseases. Compared with AAA, AS showed lower infiltration of plasma cells, follicular helper T cells, and macrophages M1, but higher infiltration of monocytes and mast cells. In addition, AS had lower expression levels of CTLA4, ICOS, TNFRSF25, and NRP but higher expression levels of TNFSF9, ICOSLG, KIR3DL1, LAIR1, TNFRSF8, IDO2, CD276, CD274, LGALS9, and TMIGD2 than AAA. KEGG metabolic pathway analysis was performed based on the expression levels of these two marker genes. GSVA analysis based on the expression levels of the two marker genes revealed significant disturbances in various metabolic and immune-related signalling pathways.

However, this study also has several limitations. Firstly, the amount of microarray data in this study is limited, which might impact the reliability and validity of our findings. However, our key findings have been consistently validated across three independent datasets, indicating their robustness and reproducibility. To further enhance the reliability of our findings, we plan to expand our experiments by including more samples in the future. Second, while we have validated the expression levels of key genes, their biological functions require further validation. To this end, several experimental approaches could be conducted, such as overexpression or knockdown of these genes in vitro or in vivo, Western blot analysis of these genes, and using cell culture or mouse models for biological validation. Furthermore, we need to further expand the sample size to verify the accuracy of the model.

In conclusion, our findings reveal a close correlation between macrophage gene expression disorder and the occurrence of AAA. Our results contribute to a comprehensive understanding of AAA development and provide new biomarkers and potential therapeutic drugs for AAA.

### Supplementary Information

Supplementary material file1. Table S1. Top 50 differentially expressed marker genes in macrophages and other immune cell types. Table S2. Primers for qPCR analyses of AAA mouse model. Table S3. Primers for qPCR analyses of AAA patients. Table S4. Details of the GEO datasets used in this study. Table S5. Details of the gene list generated in this study. Table S6. Details of the GO/KEGG enrichment terms obtained in this study. Table S7 Basic characteristics of the subjects included in this study. Figure S1. Prediction of small-molecule drugs. **A**–**H** Top 8 potential mediations with the highest absolute enrichment values.

## Data Availability

The analysis data in the article can be obtained from the GEO online database. GSE57691 was downloaded from https://www.ncbi.nlm.nih.gov/geo/query/acc.cgi?acc=GSE57691, GSE47472 downloaded from https://www.ncbi.nlm.nih.gov/geo/query/acc.cgi?acc=GSE47472. Single-cell sequencing data were obtained from https://www.ncbi.nlm.nih.gov/geo/query/acc.cgi?acc=GSE166676.

## Abbreviations

AAA: Abdominal aortic aneurysm
AS: Atherosclerosis
PAD: Peripheral artery disease
AUC: Area under the curve
CIC: Clinical impact curve
CMap: Connectivity map
CTA: CT angiography
DCA: Decision curve analyses
EVAR: Endovascular aortic repair
GO: Gene ontology
GSVA: Gene set variation analysis
KEGG: Kyoto Encyclopedia of Genes and Genome
LASSO: Least absolute shrinkage selection
PCA: Principal component analysis
RF: Random forest
ROC: Receiver operating characteristic
ssGSEA: Single-sample gene set enrichment analysis
SVM: Support vector machine
Tregs: T cell regulatory
TFs: Transcription factors
TRRUST: Transcriptional regulatory relationships revealed by sentence-based text mining
WGCNA: Weighted gene co-expression network analysis
