# Supplementary material for: Comprehensive transcriptomic analysis unveils macrophage-associated genes for establishing an abdominal aortic aneurysm diagnostic model and molecular therapeutic framework
Source: Eur J Med Res. 2024 Jun 12;29:323. doi: 10.1186/s40001-024-01900-w (PMC11167832; doi:10.1186/s40001-024-01900-w)
Supplement: Supplementary file 1 — Supplementary material file1. Table S1. Top 50 differentially expressed marker genes in macrophages and other immune cell types. Table S2. Primers for qPCR analyses of AAA mouse model. Table S3. Primers for qPCR analyses of AAA patients. Table S4. Details of the GEO datasets used in this study. Table S5. Details of the gene list generated in this study. Table S6. Details of the GO/KEGG enrichment terms obtained in this study. Table S7 Basic characteristics of the subjects included in this study. Figure S1. Prediction of small-molecule drugs. A–H Top 8 potential mediations with the highest absolute enrichment values. [file 40001_2024_1900_MOESM1_ESM.zip › supplemental Table 2.docx]

| IL-1B  CXCL1  SOCS3  SLC2A3  G0S2  CCL3  ZNF652  UBR5  GAPDH | Forward  Reverse  Forward  Reverse  Forward  Reverse  Forward  Reverse  Forward  Reverse  Forward  Reverse  Forward  Reverse  Forward  Reverse  Forward  Reverse | ATGGAAGTCTGTCTGCTCAGT TTAGGAAGACACGGATTCCAT  ATGATCCCAGCCACCCGCTCG TTACTTGGGGACACCTTTTAG  ATGGAGAGCAGCAAACATAAT TCAGGCGTTGCCAGGGGTCTC  CAATCAATGAGAGGCCGGAG  GCACCTGCTTCTGTCACCTG  ATGGAAAGTGTGCAGGAGCTG TTAAGAGGCGTGCTGCCGGAG  ATGAAGGTCTCCACCACTGCC TCAGGCATTCAGTTCCAGGTC ATGAGCTACACAGCCAGTCCT  CTAATGGTGCTGGGCGGGGGC ATGACGTCCATCCATTTCGTG  CTACACAAAACCAAAATTCTTGGTCTT GGGCTCTCTGCTCCTCCCTGT  ACGGCCAAATCCGTTCACC |
| --- | --- | --- |

**Table S2. Primers for qPCR analyses of AAA mouse**
