# Supplementary material for: Comprehensive transcriptomic analysis unveils macrophage-associated genes for establishing an abdominal aortic aneurysm diagnostic model and molecular therapeutic framework
Source: Eur J Med Res. 2024 Jun 12;29:323. doi: 10.1186/s40001-024-01900-w (PMC11167832; doi:10.1186/s40001-024-01900-w)
Supplement: Supplementary file 1 — Supplementary material file1. Table S1. Top 50 differentially expressed marker genes in macrophages and other immune cell types. Table S2. Primers for qPCR analyses of AAA mouse model. Table S3. Primers for qPCR analyses of AAA patients. Table S4. Details of the GEO datasets used in this study. Table S5. Details of the gene list generated in this study. Table S6. Details of the GO/KEGG enrichment terms obtained in this study. Table S7 Basic characteristics of the subjects included in this study. Figure S1. Prediction of small-molecule drugs. A–H Top 8 potential mediations with the highest absolute enrichment values. [file 40001_2024_1900_MOESM1_ESM.zip › supplemental Table 4.docx]

Supplementary Table 4. Details of the GEO datasets used in this study.

| **GEO accession number** | **Tissue origin** | **Data type** | **Citation** | **Description of the experiment** |
| --- | --- | --- | --- | --- |
| GSE57691 | **Abdominal aortic aneurysms (AAA) and normal abdominal aorta (AO)** | Microarray | [https://www.ncbi.nlm.nih.gov/geo/query/acc.cgi?acc=GSE57691/](https://pubmed.ncbi.nlm.nih.gov/32614923/) | Genome-wide expression analysis was conducted, comparing aortic specimens from 49 patients with abdominal aortic aneurysm (AAA) to 10 control specimens from organ donors. |
| GSE47472 | **Abdominal aortic aneurysms (AAA) and normal abdominal aorta (AO)** | Microarray | https://www.ncbi.nlm.nih.gov/geo/query/acc.cgi?acc=GSE47472 | Genome-wide expression analysis was conducted, comparing aortic neck specimens from 14 patients with abdominal aortic aneurysm (AAA) to 8 control specimens from organ donors. |
| GSE166676 | **Abdominal aortic aneurysms (AAA)** | scRNA-seq | https://www.ncbi.nlm.nih.gov/geo/query/acc.cgi?acc=GSE166676 | Four aortic tissues from aneurysmal were sequenced by 10X Genomics technology. |
